# Zebrafish as a Model System to Study the Physiological Function of Telomeric Protein TPP1

**DOI:** 10.1371/journal.pone.0016440

**Published:** 2011-02-02

**Authors:** Yiying Xie, Dong Yang, Quanyuan He, Zhou Songyang

**Affiliations:** 1 State Key laboratory for Biocontrol, Sun Yat-Sen University, Guangzhou, People's Republic of China; 2 Verna and Marrs McLean Department of Biochemistry and Molecular biology, Baylor College of Medicine, Houston, Texas, United States of America; Sun Yat-Sen University, China

## Abstract

Telomeres are specialized chromatin structures at the end of chromosomes. Telomere dysfunction can lead to chromosomal abnormalities, DNA damage responses, and even cancer. In mammalian cells, a six-protein complex (telosome/shelterin) is assembled on the telomeres through the interactions between various domain structures of the six telomere proteins (POT1, TPP1, TIN2, TRF1, TRF2 and RAP1), and functions in telomere maintenance and protection. Within the telosome, TPP1 interacts directly with POT1 and TIN2 and help to mediate telosome assembly. Mechanisms of telomere regulation have been extensively studied in a variety of model organisms. For example, the physiological roles of telomere-targeted proteins have been assessed in mice through homozygous inactivation. In these cases, early embryonic lethality has prevented further studies of these proteins in embryogenesis and development. As a model system, zebrafish offers unique advantages such as genetic similarities with human, rapid developmental cycles, and ease of manipulation of its embryos. In this report, we detailed the identification of zebrafish homologues of TPP1, POT1, and TIN2, and showed that the domain structures and interactions of these telosome components appeared intact in zebrafish. Importantly, knocking down TPP1 led to multiple abnormalities in zebrafish embryogenesis, including neural death, heart malformation, and caudal defect. And these embryos displayed extensive apoptosis. These results underline the importance of TPP1 in zebrafish embryogenesis, and highlight the feasibility and advantages of investigating the signaling pathways and physiological function of telomere proteins in zebrafish.

## Introduction

Vertebrate telomeres are composed of duplex telomeric DNA repeats with 3′ single-stranded overhangs. Along with the telomerase, a multitude of proteins participate in telomere regulation. For example, six core telomeric proteins TRF1, TRF2, RAP1, TIN2, POT1 and TPP1 can form a large molecular weight complex – telosome/shelterin – that localize to the telomere chromatin for telomere length maintenance and end protection [1], [2], [3]. Using proteomic approaches that isolate telomeric protein complexes, we identified human TPP1 as an important regulator of telomeres, and found it to directly interact with two other telosome components POT1 and TIN2 through distinct domain structures on these proteins [4], [5]. Furthermore, such interactions are critical for TPP1 function and telosome assembly [4], [5]. Expression of mutants of TPP1 that failed to interact with TIN2 or POT1 led to DNA damage responses at the telomeres and telomere length dysregulation [4], [6], [7], [8]. Similar results were also obtained when TPP1 levels were inhibited by RNAi [7], [9], [10], [11].

The physiological function of telosome components has also been examined in mice. Homozygous inactivation of TRF1, TRF2, POT1 or TIN2 in mice led to early embryonic lethality (∼E6–7) [12], [13], [14], [15], [16], [17], suggesting that the telosome may play a critical role during embryonic development. TPP1 knockout mice die perinatally [18]. A spontaneous autosomal recessive mutation in the mouse TPP1 gene has also been found (*acd* mice) [19]. This hypomorphic mutation occurs in the intronic region of the TPP1 gene and results in aberrant mRNA splicing [19]. The *acd* mice typically die within 1–2 days after birth. The embryos have striking defects in caudal specification, limb patterning and axial skeleton formation. This suggests that TPP1, in addition to serving as a telomere regulator, may also play important roles in development. Consistent with this idea, developmental genes such as Wnt3a, Dll1 and Fgf8 displayed altered expression in *acd* mice [19].

While *in vivo* studies in mice and other model organisms have offered important clues to the physiological function of telomeric proteins, premature death and early embryonic lethality preclude detailed analysis of the cell signaling events mediated by these proteins in development, particularly during early embryogenesis. Of the many model organisms, zebrafish (*Danio rerio*) provides unique advantages for studying gene function and vertebrate development, such as significant similarities with humans in genetic information and physiology, rapid *ex utero* embryo development and reproductive cycles, and the ease with which the large and transparent embryos may be manipulated and observed. In fact, several models of human diseases have been developed in zebrafish [20], [21].

It has been shown that zebrafish telomere sequences are very similar to human telomeres in both length and repeat sequences [22], [23]. And the zebrafish telomerase was cloned and demonstrated to be necessary for hematopoiesis [24]. However, the cell signaling pathways important for telomere regulation in zebrafish remain to be elucidated.

We report here the identification of the zebrafish (zf) homologue of human TPP1. We showed that zfTPP1 could localize to telomeres, and its domain structure and interaction with other telosome components such as POT1 and TIN2 was conserved. Furthermore, knocking down zfTPP1 by morpholino antisense oligos led to developmental defects including neural death, heart malformation and caudal defect. These results indicate that TPP1 plays an important role in zebrafish embryogenesis and that zebrafish presents a good system to study the physiological function of telomeric proteins.

## Materials and Methods

### Zebrafish maintenance

Wild-type zebrafish (strain AB) were raised and maintained in E3 buffer (5 mM NaCl, 0.17 mM KCl, 0.33 mM CaCl_2_, 0.33 mM MgSO_4_, and 0.00001% Methylene Blue) under standard conditions (28.5°C and 14 hr/10 hr of light/dark cycles) [25]. Embryo staging includes hours post fertilization (hpf) and morphological attributes [25]. Animal care was in accordance with institutional guidelines, and the animal protocol was approved by the Institutional Animal Care and Use Committee of Baylor College of Medicine (Assurance number 3823-01) in accordance with the Guide for the Care and Use of Laboratory Animals published by the US National Institutes of Health (NIH Publication No. 85–23, revised 1996).

### Morpholino antisense oligonucleotides

Morpholino antisense oligonucleotides (MO) were obtained from Open Biosystems (Table 1), and used at a concentration of 1–2 µg/µl in injection buffer (29 mM NaCl, 0.35 mM KCl, 0.2 mM MgSO_4_, 0.3 mM CaCl_2_, 2.5 mM HEPES pH 7.6). Approximately 1–6 ng of each MO was injected into zebrafish embryos at 1–2 cell stage.

**Table 1:** Morpholino oligonucleotides used in this study.

MO name	MO type	MO sequence
zfTPP1-MO1	Translational blocker (ATG)	GACGTTTCATTTTAGGTGGATTCAT
zfTPP1-MO1-mu	Translational blocker (ATG)	GACCTTTGATTTAAGCTGCATTCAT
zfTPP1-MO2	Translational blocker (UTR)	ACTGGTGTAAATGCAGAAAAGAGC
Chordin-MO	Translational blocker	ATCCACAGCAGCCCCTCCATCATCC

MO, morpholino. Mu, mutation.

### Constructs and cell lines

Full-length cDNAs encoding zebrafish (zf) TPP1, TIN2 and POT1 (GenBank accession numbers HQ652075 and HQ652076) were obtained from a zebrafish cDNA library (Open Biosystems) by PCR amplification, and subsequently cloned into pENTR-TOPO vectors (Invitrogen). Primer sequences are listed in Table 2. FLAG-, TAP- or V5-tagged full-length human TPP1 (hTPP1) or zfTPP1 were cloned into pCL-based retroviral vectors. The retroviral vectors were transfected into BOSC23 cells for packaging [2]. Viruses were collected 48 hours after transfection and used to infect the zebrafish cell line ZF4 [26] to generate stable cells. zfTPP1, zfPOT1, and zfTIN2 were also cloned into pDEST27 (Invitrogen) for the expression of GST-fusion proteins in mammalian cells. For zfTIN2 homologues, the N-terminal regions (zfTIN2N1, amino acid 2–225; N2, amino acid 2–210; N3, amino acid 2–257) were cloned into pDEST27.

**Table 2:** Primers used to identify zfTPP1, zfTIN2 and zfPOT1.

Primer name	Sequence
zfTPP1-5′	GGCGCGCCAATGAATCCACCTAAAATGAAACGTCG
zfTPP1-3′	AACTGAACCCGCTTCAGCATTGATATC
zfTIN2-1-5′	CACCATGAACAGGAAAACAAAAACAGCGACTAGATCT
zfTIN2-1-3′	ATGTCTGCTTCCAGGAGATGAAGAGGGAC
zfTIN2-1N-3′	GCCGAGGTGTCCAAGGCATACTTCATGCT
zfTIN2-2N-5′	CACCATGGAAGCGCTGTGGACGAACAAAATCGAAGAC
zfTIN2-2N-3′	TGTGTGGTGTTCAAGAAGACTCTTCATATCC
zfTIN2-3N-5′	CACCATGTGGCAGGTGCTGCAGCACAAGAGCCTGG
zfTIN2-3N-3′	CTCCTCCTCCTGCATTGGCTCCATCAG
zfPOT1-5′	CACCATAATCAAAGGCATGCCTGTAGAAAAAGTCAACAATTCA
zfPOT1-3′	TTGTTTAACAATTTCTGTGTTTGTGATCTGGTAACAAAC
zfPOT1C-5′	CACCATGGACGGGACCAGGTGTCAGCTTCCCTTGT

### Immunoprecipitation and western blotting analysis

For interaction studies, 293T cells [2] were co-transfected with the appropriate constructs encoding various proteins using Lipofectamine^2000^ (Invitrogen). At 48 hours post transfection, the cells were harvested and lysed with NETN (20 mM Tris, pH 8, 1 mM EDTA, 100 mM NaCl, and 0.5% NP-40). The extracts were then incubated for 1 hour at 4°C with anti-FLAG M2 agarose beads (Sigma) or GSH beads (GE). The bound proteins were eluted in 2x Laemmli buffer, resolved by SDS-PAGE, and western blotted with the indicated antibodies. The antibodies used were anti-FLAG M2 HRP (Sigma), anti-V5 HRP (Bethyl Laboratories), and anti-GST HRP (Roche).

### Telomere chromatin immunoprecipitation

Parental ZF4 cells or those expressing zfTPP1-TAP were crosslinked with 1% formaldehyde for 10 min at room temperature, rinsed twice with ice-cold 1xPBS, and incubated with 2 M Glycine to stop crosslinking. The cells were subsequently collected using a silicon scraper, lysed in lysis buffer (50 mM Hepes, pH 7.5, 150 mM NaCl, 0.1% SDS, 0.1% sodium deoxycholate, and 1% Triton X-100), and sonicated (Virsonic 600) at 4°C. Each sample was pre-cleared with 50 µl agarose beads plus 5% BSA and sheared salmon sperm DNA (5 µg). The supernatant was then incubated with 20 µl of IgG beads (Amersham) plus BSA (5%) and sheared salmon sperm DNA (25 µg) for 2 hours at 4°C. Beads were washed sequentially with the following buffers: buffer I (50 mM Hepes, pH 7.5, 150 mM NaCl, 2 mM EDTA, 0.1% SDS, and 1% Triton X-100), buffer II (buffer I containing 500 mM NaCl), buffer III (10 mM Tris, pH 8.0, 0.25 M LiCl, 1 mM EDTA, 0.5% NP-40, 0.5% sodium deoxycholate, and 1 mM DTT), and buffer IV (10 mM Tris, pH 8.0, 1 mM EDTA, and 1 mM DTT). The bound complexes were eluted and incubated overnight at 65°C to reverse crosslinking. Immunoprecipitated DNA and input DNA were dot-blotted onto nitrocellulose membranes and analyzed by southern blot using the radiolabelled (TTAGGG)_3_ probe as described previously [7].

### Acridine Orange staining of zebrafish embryos

Live embryos were dechorionated with forceps and then incubated in E3 buffer containing 5 µg/ml acridine orange (Sigma) at room temperature for 10 minutes as described [27]. The stained embryos were then washed with E3 buffer, and visualized under a fluorescent microscope (Axioplan 2 Imaging, Zeiss).

## Results

### The TPP1 homologue in zebrafish associates with telomeres

**Figure 1:**
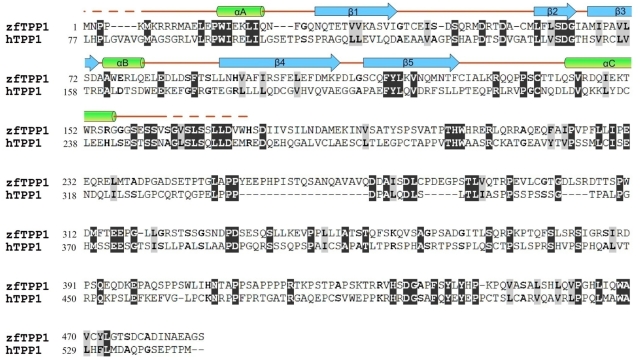
Sequence alignment of zebrafish TPP1 (zfTPP1) and human TPP1 (hTPP1). Alignment was carried out by ClustalW. The conserved residues are in shaded black and gray. The alpha helices (cylinders) and beta strands (arrows) of the hTPP1 OB-folds are illustrated above the aligned sequences.

Homologues of many human telomere proteins have been identified in lower organisms that have proven to be important model systems for studying telomere biology. Sequence and structural comparisons suggest that zebrafish possess homologues of all six components of the human telosome (data not shown). Further analysis revealed that the gene for zebrafish TPP1 (zfTPP1) is located on chromosome 7. We subsequently cloned the cDNA for zfTPP1, and found it to encode a polypeptide of 487 amino acids that shares extensive sequence and structural similarities with human TPP1 (hTPP1) (Fig. 1, 2A). Importantly, zfTPP1 appears to contain all the domains important for hTPP1 interaction with TIN2 and POT1, including the N-terminal OB fold, the POT1-binding RD domain, and the C-terminal TIN2-interacting domain (TID).

**Figure 2:**
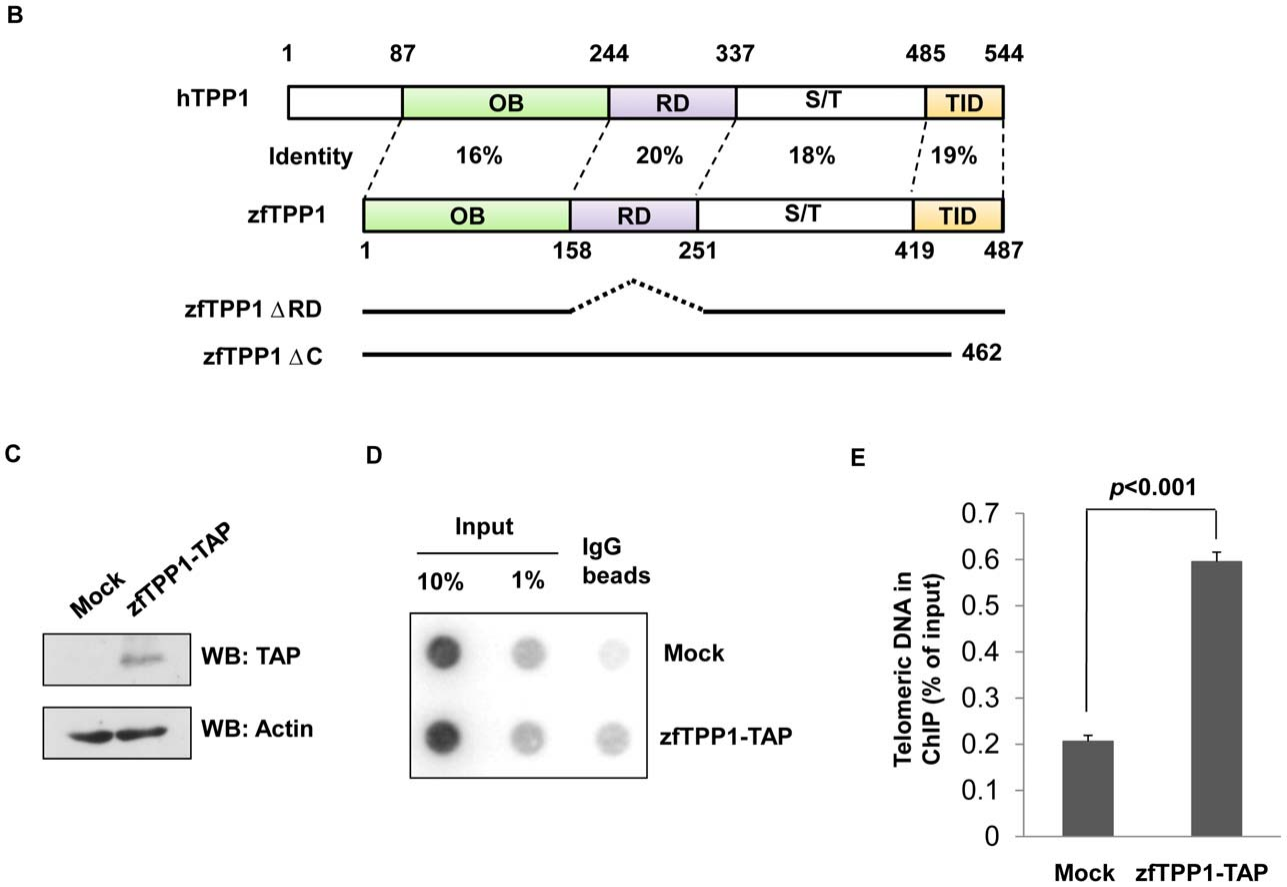
Characterization of zfTPP1. (A) Schematic representation of domain organization of human and zebrafish TPP1. OB, oligonucleotide/oligosaccharide binding fold. RD, POT1 recruitment domain. S/T, Serine-rich region. TID, TIN2-interacting domain. (B) Expression of TAP-tagged zfTPP1 in zebrafish cells. Whole cell extracts from parental and TAP-tagged zfTPP1 expressing zebrafish cell line ZF4 were western blotted with peroxisome-conjugated protein A. Actin was used as a loading control. (C) Telomere association of zfTPP1. Parental and TAP-tagged zfTPP1 expressing ZF4 cells were crosslinked and used for chromatin immunoprecipitation with protein A beads. The precipitated DNA was analyzed by southern blotting. (D) Quantification of data in D. Error bars indicate standard error (n = 3).

The conservation of domain structures in zfTPP1 suggests its possible role in regulating zebrafish telomeres. To further probe this idea, we first sought to determine whether zfTPP1 could localize to telomeres in zebrafish. Because antibodies against zfTPP1 are unavailable, we examined the targeting of ectopically expressed zfTPP1. Zebrafish ZF4 cells stably expressing TAP-tagged zfTPP1 were generated through infection with VSVG pseudo-typed retroviruses (Fig. 2B). Chromatin immunoprecipitation (ChIP) experiments were subsequently carried out. As shown in Figure 2C and 2D, telomere sequences were enriched in IgG pull-down samples from TAP-tagged zfTPP1 expressing cells, indicating that zfTPP1 can associate with telomere DNA in zebrafish cells. The amount of telomeric DNA that was brought down by zfTPP1 appeared low, possibly due to lower expression of zfTPP1-TAP in these cells.

### Interactions between zfTPP1 and zfPOT1

**Figure 3:**
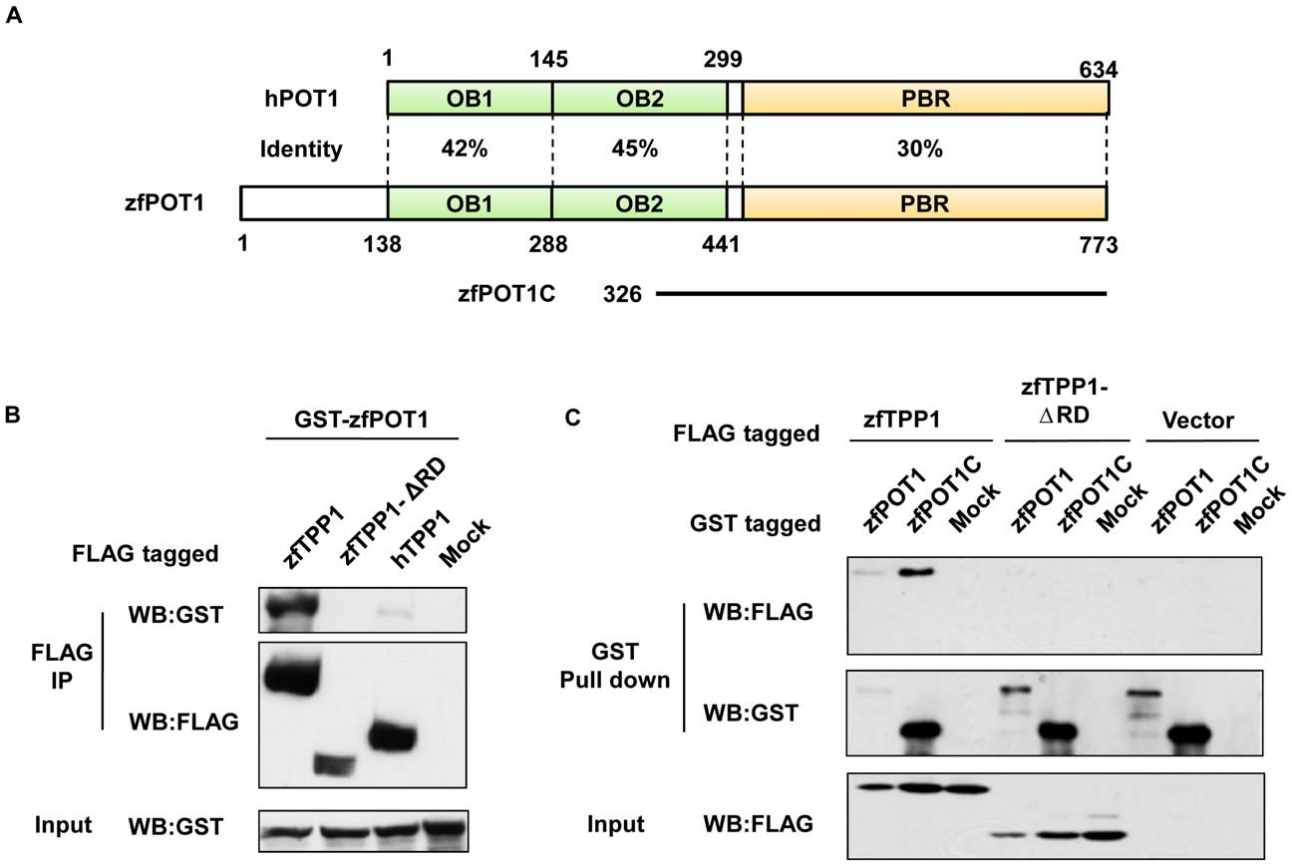
zfTPP1 interacts with zfPOT1. (A) Schematic representation of domain homology between human and zebrafish POT1. PBR, TPP1-binding region. (B) The RD domain is important for zfTPP1-zfPOT1 interaction. Extracts from 293T cells co-expressing GST-tagged zfPOT1 with FLAG-tagged full-length zfTPP1, zfTPP1-ΔRD (RD domain deletion mutant), or hTPP1 were immunoprecipitated with anti-FLAG antibodies, and analyzed by western blotting with the indicated antibodies. (C) The C-terminal region of POT1is important for its interaction with zfTPP1. Extracts from 293T cells co-expressing FLAG-tagged zfTPP1 or zfTPP1-ΔRD with GST-tagged full-length zfPOT1 or zfPOT1 C terminal domain (zfPOT1C) were immunoprecipitated with anti-GST antibodies and analyzed by western blotting with the indicated antibodies.

In human cells, the interaction between TPP1 and POT1 is mediated by the TPP1 RD domain and the POT1 PBR domain [4]. To determine whether such interactions also occur in zebrafish, we first cloned the cDNA for zebrafish POT1 (zfPOT1). As in the case of humans, the zebrafish genome contains only one POT1-like gene, whose product exhibits high homology to human POT1 (Fig. 3A). Moreover, zfPOT1 also harbors two N-terminal OB folds and a C-terminal domain similar to the TPP1 binding domain (PBR) of human POT1.

The interaction between zebrafish TPP1 and POT1 was examined in 293T cells. GST-tagged full-length zfPOT1 was co-expressed with FLAG-tagged full-length zfTPP1 and TPP1 RD domain deletion mutant. Consistent with previous findings in human cells [4], the interaction between zebrafish POT1 and TPP1 also required the RD domain (Fig. 3B, C), as zfTPP1-ΔRD failed to co-immunoprecipitate with zfPOT1. Similarly, the C-terminal PBR domain of POT1 was much more efficient at co-precipitating with zfTPP1 (Fig. 3C) [4]. These data indicate that zfTPP1 interacts with zfPOT1 through their respective RD and PBR domains.

### Interactions between zfTPP1 and zfTIN2

**Figure 4:**
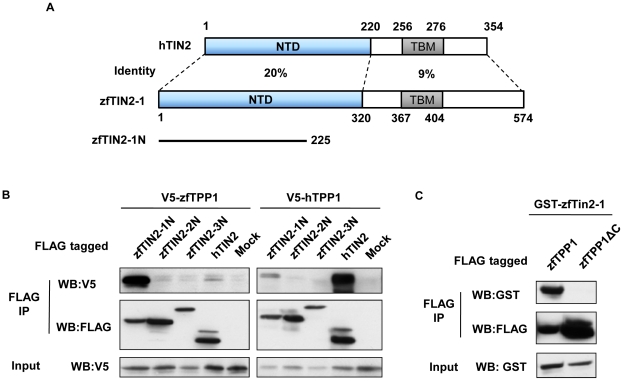
zfTPP1 interacts with zfTIN2-1 through its C-terminus. (A) Schematic representation of domain homology between human and zebrafish TIN2. NTD, N-terminal domain. TBM, TRFH-binding motif. (B) zfTPP1 interacts with zfTIN2-1. Extracts from 293T cells co-expressing V5-tagged zfTPP1 or hTPP1, together with FLAG-tagged N terminal part of zfTIN2-1, zfTIN2-2, or zfTIN2-3, were immunoprecipitated with anti-FLAG antibodies and western blotted. FLAG-tagged hTIN2 was also included. (C) The C terminal domain of zfTPP1 mediates its interaction with zfTIN2-1. Extracts from 293T cells co-expressing GST-tagged full-length zfTIN2-1 with FLAG-tagged full-length zfTPP1 or zfTPP1-ΔC were analyzed by immunoprecipitation and western blotting.

To identify zebrafish TIN2 homologues, we utilized a zebrafish cDNA library. Interestingly, we were able to clone three potential TIN2 homologues that we named zfTIN2-1, zfTIN2-2, and zfTIN2-3. The three putative TIN2 homologues share an N-terminal domain that is homologous to hTIN2, but harbor different C-terminal sequences. It should be noted that only zfTIN2-1 contains a putative TRFH-binding-motif (TBM) [28], [29] (Fig. 4a).

The N-terminal region of TIN2 has been shown to interact with TPP1 [8]. We therefore co-expressed zfTPP1 with the N-terminal regions of the three candidate zebrafish homologues in 293T cells to examine their interactions with TPP1. As shown in Figure 4b, only the N-terminal region of zfTIN2-1 was able to co-immunoprecipitate with zfTPP1. Taken together with our sequence analysis data, these findings indicate that zfTIN2-1 is the zebrafish ortholog of hTIN2 (and hitherto referred to as zfTIN2). Interestingly, zfTIN2 is located on chromosome 7 and in the vicinity of the zfTPP1 locus, raising the possibility of co-evolution of these two genes. Furthermore, deletion of the C-terminal TIN2 interaction domain (TID) on zfTPP1 (amino acids 462–487) abolished its interaction with zfTIN2 (Fig. 4c), indicating the importance of the TID domain in mediating TIN2-TPP1 interaction in zebrafish. It is also interesting to note that hTPP1 could interact weakly with zfTIN2 and vice versa (Fig. 4b), further highlighting the evolutionary conservation of important domains/residues for telomere protein interactions.

Collectively, our results demonstrate that zfTPP1 can localize to telomeres and interact with zfPOT1 and zfTIN2, as is the case in human cells. These findings suggest that the subunits and function of the telosome may be conserved between zebrafish and human, and provide the basis for using zebrafish as a model organism to study the physiological function of telosome subunits.

### Knockdown of zfTPP1 by morpholino oligonucleotides resulted in developmental defects

In zebrafish, the function of genes can be assessed through morpholino-mediated knockdown [30]. For example, morpholino antisense oligonucleotides are often designed to bind near the translation initiation sites or splicing junctions on mRNA sequences of the target gene, thereby inhibiting the expression of morpholino-targeted genes. This block of expression does not occur through target mRNA degradation, and therefore cannot be assessed by examining mRNA levels. Morpholino oligos may be injected into zebrafish embryos at different stages to determine how disrupting expression of a particular gene may affect zebrafish development [31].

**Figure 5:**
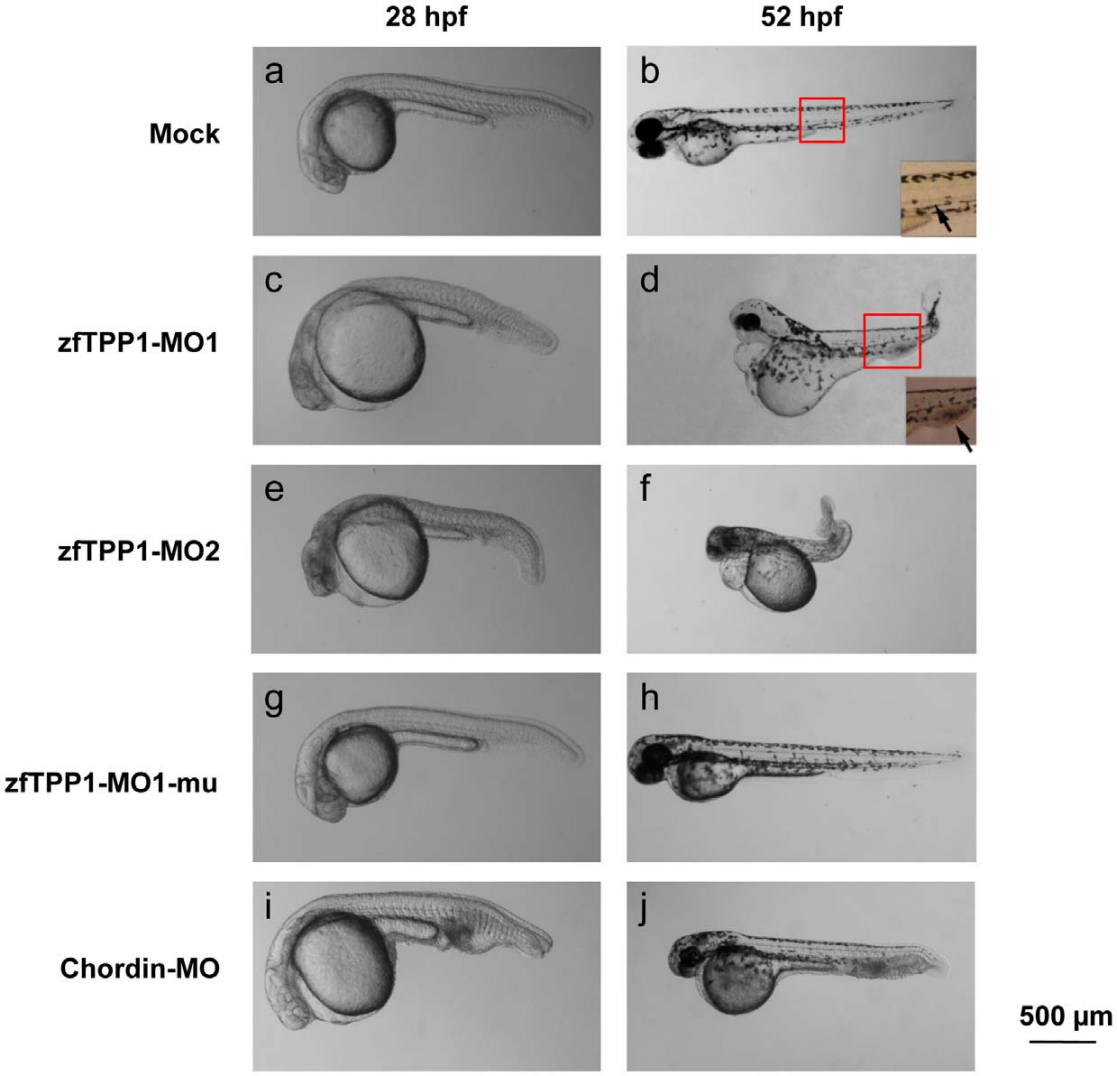
Expression of zfTPP1 morpholinos leads to defects in embryonic development. Mock injected zebrafish embryos (a, b) and those injected with zfTPP1 MO1 (6 ng) (c, d) and MO2 (3 ng) (e, f), zfTPP1 MO1 mutant (6 ng) (g, h), or Chordin MO (6 ng) (i, j), were observed under the microscope at 28 and 52 hour post fertilization (hpf). Magnified images of the trunk region of mock and MO1 injected embryos were also included. Arrows indicate blood in the embryos. Scale bar, 500 µm.

To investigate the cell signaling pathways of TPP1, we utilized morpholino oligonucleotides against TPP1 to knockdown TPP1 in zebrafish embryos (Fig. 5). Four different morpholino oligos (25-mer) were generated for the experiment (Table 1). MO1 targets the translational initiation site of the zfTPP1 mRNA, while MO2 is against the 5′UTR region of zfTPP1 mRNA. These two oligos are expected to block zfTPP1translation. MO1-mu targets the same region as MO1 but carries five missense mutations, and therefore serves as a control for the specificity of MO1. Chordin-MO is against the development regulator Chordin, and serves as a positive control for morpholino knockdown. Zebrafish embryos at one or two-cell stage were injected with the morpholino oligos and then allowed to further develop for another 28–52 hours before analysis.

Consistent with published reports, injection of Chordin-MO led to U-shaped somites and an abnormal tail fin with multiple folds (Fig. 5i, 5j) [30]. In comparison, introduction of either MO1 or MO2 morpholinos resulted in severe developmental defects in zebrafish embryos (Fig. 5c-f). The defects caused by MO1 and MO2 appeared similar. Notably, MO1-mu injection did not lead to any overt phenotypical changes (Fig. 5h), suggesting that loss of zfTPP1 activity was responsible for the defects caused by MO1 and MO2. A lower dose of MO2 (3 ng) was needed to produce these phenotypes compared to MO1 (6 ng), a likely result of differences in knockdown efficiencies between these two morpholinos.

**Figure 6:**
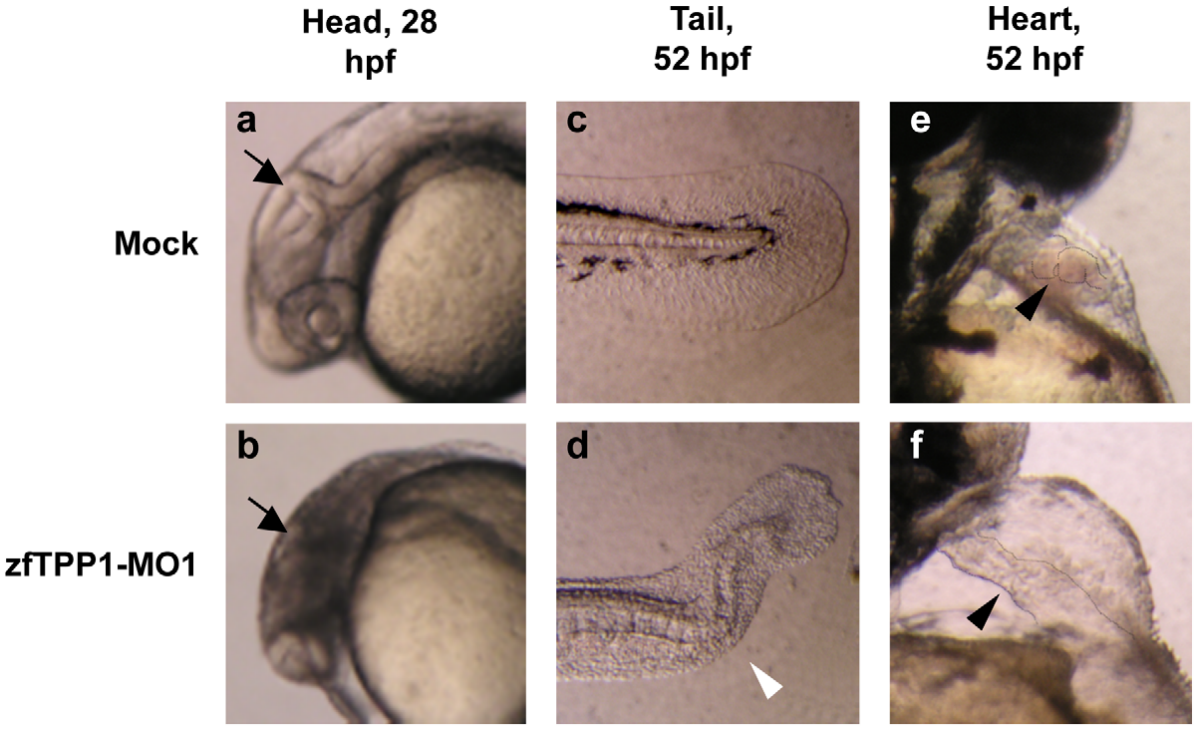
zfTPP1 knockdown results in defects in the brain, tail and heart. Mock (a, c, e) and zfTPP1-MO1 injected embryos (b, d, f) were visualized at the indicated time points. In a and b, arrows point to the brain. In c and d, arrowhead indicates a curly tail. In e and f, arrowheads point to the heart.

At 28 hours post fertilization (hpf), mock injected or MO1-mu injected embryos developed clear heads with easily identifiable brain structures (Fig. 6a). In contrast, most zfTPP1 morphants (MO1 or MO2 injected embryos) (∼90%) developed a dense and opaque head (Fig. 6b), suggesting defects in neural development. At 52 hpf, mock injected or MO1-mu injected embryos exhibited straight torsos with somites (Fig. 5b, 6c). However, around 80% of zfTPP1 morphants developed curly tails (Fig. 6d), suggesting a defect in caudal development. Interestingly, a similar caudal defect phenotype was also observed in *acd* mice that carry a *TPP1* mutation [19].

During normal zebrafish embryogenesis, a simple heart tube composed of the myocardium (outer muscular layer) and the endocardium (inner endothelial layer) emerges by 24 hpf. It then transforms into two morphologically distinct chambers with the linear heart tube bending gradually at the boundary between the two chambers to create an S-shaped loop [32]. At 52 hpf, the heart of the mock injected embryos clearly formed a loop with two chambers and contracted at a rate of ∼180 beats/minute, driving normal blood flow (Fig. 6e). In MO1 injected embryos, on the other hand, looping appeared incomplete and remained central and linear (Fig. 6f). Moreover, pericardial oedema – enlargement of the heart cavity (space surrounding the heart) – was very apparent (Fig. 6f). We observed low cardiac contractility (∼50 beats/minute) and strikingly, little blood flow through the heart (Fig. 6f). Consequently, blood became pooled elsewhere in the morphant body (Fig. 6d). Taken together, these observations point to profound defects in the cardiovascular development of the zfTPP1 morphants.

### zfTPP1 morphants display excessive apoptosis

In multicellular organisms, early development is a tightly controlled balance between cell proliferation, differentiation, and apoptosis. Given the important function of telomeric proteins in maintaining genome stability, we reasoned that the defects in zfTPP1 morphants could be a result of dysregulated progenitor cells or apoptosis.

**Figure 7:**
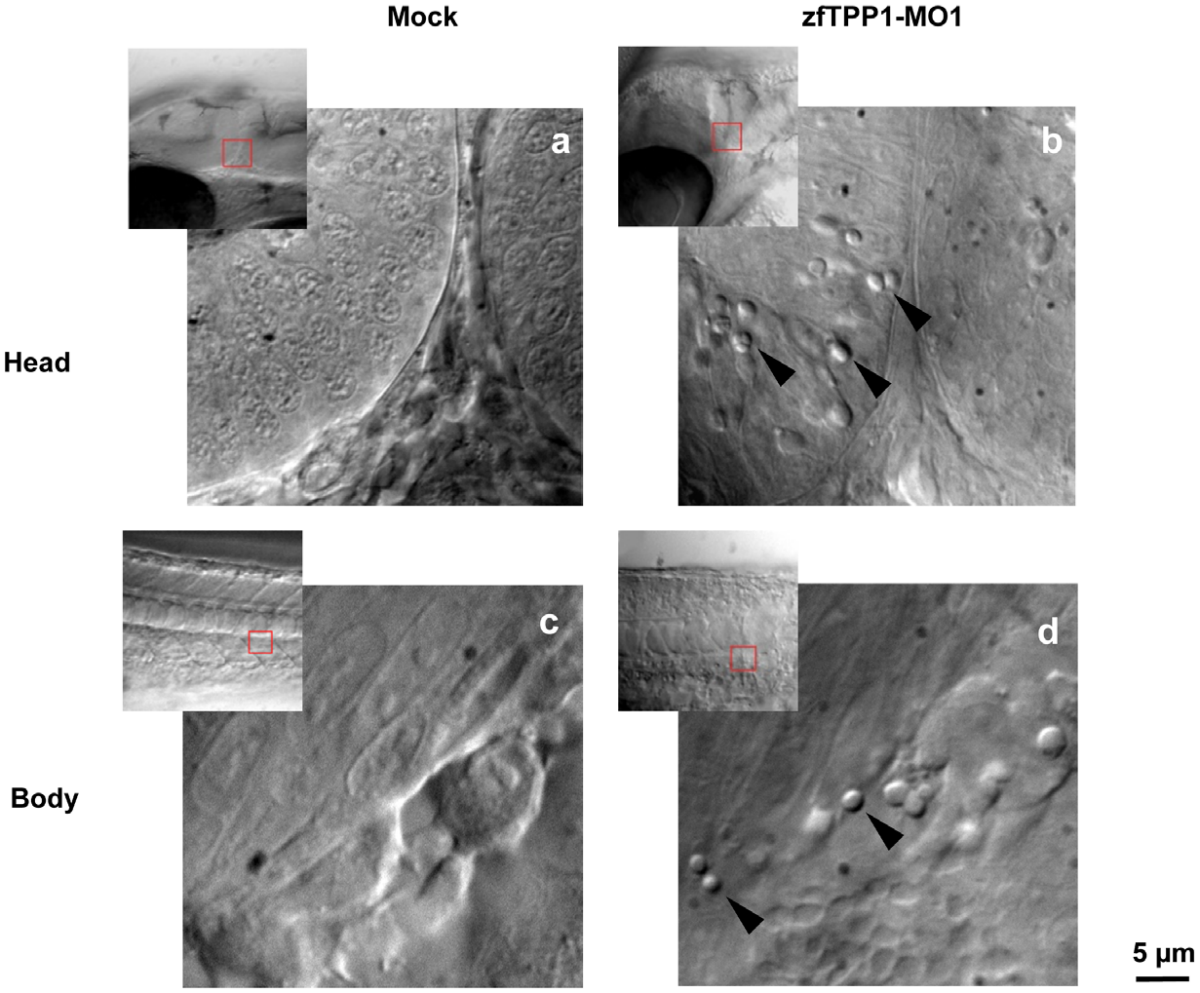
zfTPP1 knockdown leads to accumulation of button-like structures. Nomarski DIC images of regions of the head (a, b) and body (c, d) of mock and zfTPP1 MO1 (6 ng) injected embryos were obtained at 28 hpf. Button-like structures are indicated by arrows. Scale bar, 5 µm.

Upon close examination of the zfTPP1 morphants under Nomarski DIC optics, we observed many button-like objects in MO1 injected embryos (Fig. 7). We hypothesized that these objects might in fact be apoptotic cells and tested this idea using acridine orange, a vital dye that specifically stains apoptotic cells through its direct binding to nucleic acid [33].

**Figure 8:**
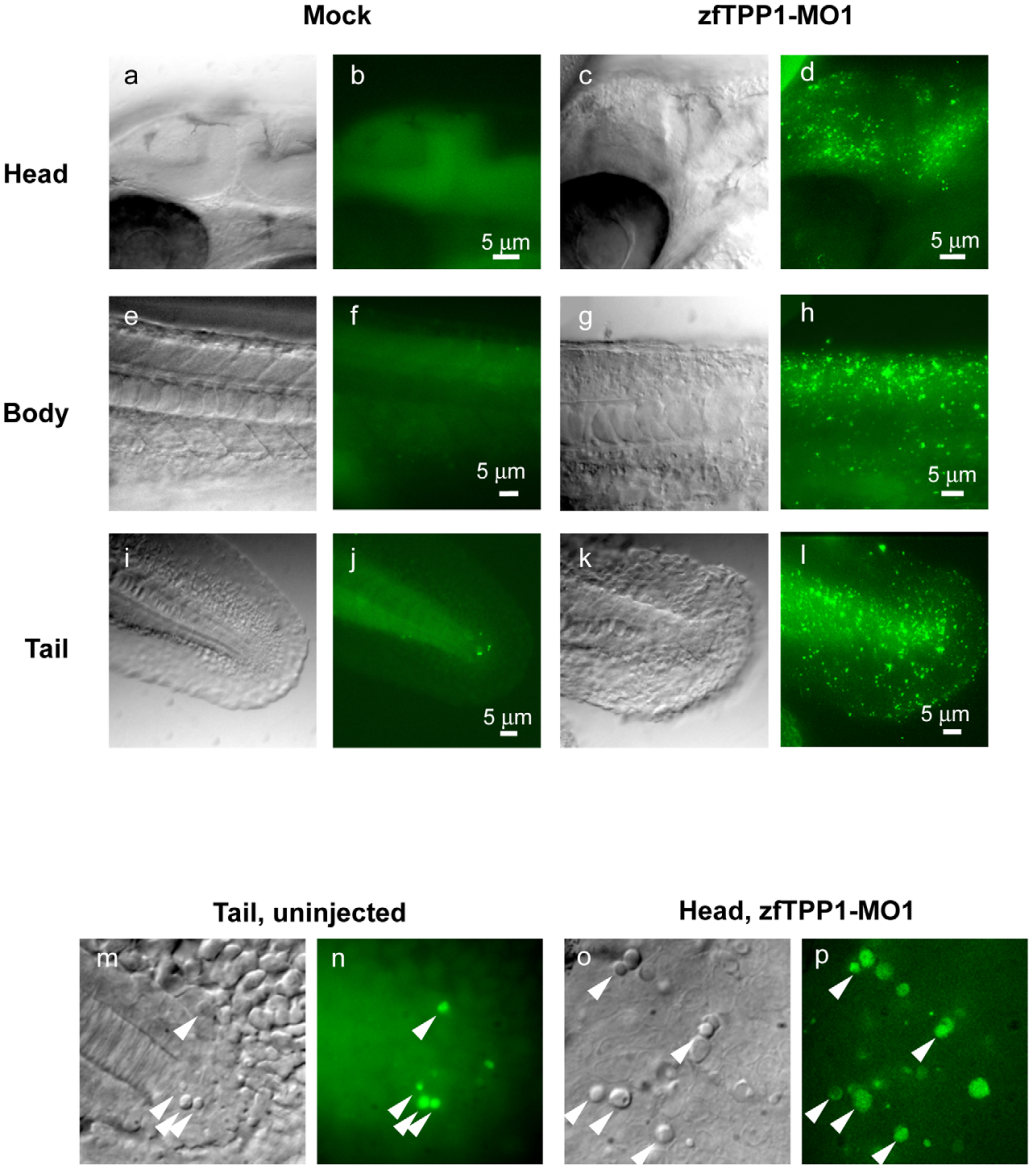
zfTPP1 knockdown induces apoptosis. Embryos injected with zfTPP1 MO1 were stained with acridine orange and visualized (at 28hpf) under Nomarski DIC and fluorescent microscopes (scale bar, 5 µm). Magnified Nomarski DIC and fluorescent images (28 hpf) of the tail (mock injected) (m, n) and head region (zfTPP1 MO1 injected) (o, p) of zebrafish embryos. Arrowheads indicate button-like structures.

At 28 hpf, differences in acridine orange staining patterns between mock injected embryos and zfTPP1 morphants became quite pronounced (Fig. 8). The mock injected embryos generally exhibited no staining (Fig. 8b, 8f). In the tail region where normal developmental apoptosis occurs at this stage for shaping tails, a few acridine orange stained cells could be observed (Fig. 8j). These stained cells correlated with the button-like objects observed under Nomarski optics (Fig. 8), suggesting that the buttons were indeed apoptotic cells. In contrast, the zfTTP1 morphants exhibited extensive staining throughout the body (Fig. 8d, 8h, 8l). The pattern also correlated with the button-like objects we observed (Fig. 8o, 8p), suggesting excessive apoptosis in zebrafish embryos knocked down for TPP1 and that extensive apoptosis may be responsible for the phenotypes of zfTPP1 morphants.

## Discussion

In this study, we reported the identification of zebrafish homologues of human TPP1, POT1, and TIN2. We presented evidence that zfTPP1 could localize to the telomeric chromatin, and demonstrated the interactions between zebrafish TPP1 and its partners TIN2 and POT1. For example, we showed that the C-terminal region of zfPOT1 was responsible for mediating its interaction with zfTPP1. And the C-terminal tail and putative RD domain in zfTPP1 were essential for its interaction with zfPOT1 and zfTIN2. Such mutational analysis indicates that the domains utilized to mediate interactions between these proteins in zebrafish are similar to those in mammalian cells. These observations indicate that the components and organization of the telosome are conserved from zebrafish to human, providing the molecular basis for using zebrafish to understand the function and signal transduction of human telomeric proteins.

Compared to mice, zebrafish represents an attractive alternative *in vivo* model. The telomere length of laboratory inbred mice is extremely long (∼50–150 kb) [34], [35], whereas the length of zebrafish telomeres (∼4–20 kb) is much closer to that of human telomeres (∼8–10 kb) [23], [24], [36]. Furthermore, the availability of large amounts of embryos within a short amount of time and the easily observable embryogenesis and organogenesis processes make zebrafish ideal tools for studying genes whose disruption in mice may lead to embryonic lethality.

Mice homozygous knockout for TPP1 die perinatally [18]. The *acd* mice carrying a hypomorphic mutation in the TPP1 gene show reduced TPP1 expression and phenotypes including urogeneital developmental defects and caudal truncation [19], [37], [38]. zfTPP1 knockdown by morpholinos also led to the death of zebrafish embryos. In this case, however, we were able to document the morphological changes that occurred with TPP1 inactivation during embryogenesis. Defects in multiple systems were observed including neural development and caudal specification, phenotypes that are strikingly similar to those observed in *acd* mice.

In zfTPP1 morphants, we also observed incomplete heart development with associated progressive pericardial edema, low cardiac contractility, and blood pooling. Defects in cardiovascular development have not been described in *acd* mice. Previous studies have indicated that cardiac myocytes undergo continuous renewal, where senescent and poorly contracting myocytes are replaced with younger and more efficient cells [39]. Old myocytes undergo cellular senescence while new myocytes can derive from cardiac stem or progenitor cells. It is interesting to note that old myocytes appear to have much shorter telomeres compared to young myocytes [39], [40], [41], suggesting that telomere maintenance pathways may play a role in this process. In addition, the ability of cardiac progenitor cells to divide might also be under the regulation of the telomerase and telomeric proteins. To date, TPP1 is the only protein in the telosome found to directly interact with and recruit the telomerase [7], [42]. One possible explanation for the heart defects in zfTPP1 knockdown embryos is the reduced ability of cardiac progenitor cells to produce new myocytes. As a result, both the number and vigor of cardiac myocytes are greatly compromised. This idea is consistent with the poor contraction we observed in zfTPP1 knockdown embryos. Alternatively, the cardiomyocytes in zfTPP1 knockdown embryos may undergo accelerated senescence. Consequently, newly formed cardiac myocytes are not sufficient to fulfill all the functional requirement of the cardiovascular system. The role of zfTPP1 in heart development warrants future investigation.

zfTPP1 loss of function appeared to trigger excessive apoptosis in zebrafish embryos as well. While off-target effects cannot be completely ruled out, the fact that two independent morpholino oligos that target different steps during TPP1 expression argues against this possibility. In mammalian cells, TPP1 knockdown leads to DNA damage responses at the telomeres, where multiple DNA damage response proteins are recruited to the telomere ends [7]. In fact, disruption of the telosome complex through inhibition of its subunit in general leads to DNA damage response at the telomeres and p53 activation [3], [43]. The skin hyperpigmentation and hair growth defects in TPP1 deletion mice can be rescued by p53 deficiency [18]. The caudal dysgenesis in *acd* mice is also dependent on p53 pathway [38]. These data suggesting that the involvement of apoptosis in TPP1 deficiency is conserved across different species.
