# Correction: Zebrafish as a Model System to Study the Physiological Function of Telomeric Protein TPP1

**DOI:** 10.1371/annotation/21d9bc69-ccac-49b8-82f2-a6303e93618a

**Published:** 2011-02-17

**Authors:** Yiying Xie, Dong Yang, Quanyuan He, Zhou Songyang

The 2nd affiliation for the 4th author was not indicated. Zhou Songyang is affiliated with both 1 State Key laboratory for Biocontrol, Sun Yat-Sen University, Guangzhou, People's Republic of China, and 2 Verna and Marrs McLean Department of Biochemistry and Molecular biology, Baylor College of Medicine, Houston, Texas, United States of America.

